# Fault Recognition of Rolling Bearings Based on Parameter Optimized Multi-Scale Permutation Entropy and Gath-Geva

**DOI:** 10.3390/e23081040

**Published:** 2021-08-13

**Authors:** Haiming Wang, Qiang Li, Shaopu Yang, Yongqiang Liu

**Affiliations:** 1 School of Mechanical, Electronic and Control Engineering, Beijing Jiaotong University, Beijing 010044, China; whm@stdu.edu.cn; 2 State Key Laboratory of Mechanical Behavior and System Safety of Traffic Engineering Structures, Shijiazhuang Tiedao University, Shijiazhuang 050043, China; yangsp@stdu.edu.cn (S.Y.); liuyq@stdu.edu.cn (Y.L.)

**Keywords:** rolling bearing, fault recognition, parameter optimized multi-scale permutation entropy, skewness, GG fuzzy clustering

## Abstract

To extract fault features of rolling bearing vibration signals precisely, a fault diagnosis method based on parameter optimized multi-scale permutation entropy (MPE) and Gath-Geva (GG) clustering is proposed. The method can select the important parameters of MPE method adaptively, overcome the disadvantages of fixed MPE parameters and greatly improve the accuracy of fault identification. Firstly, aiming at the problem of parameter determination and considering the interaction among parameters comprehensively of MPE, taking skewness of MPE as fitness function, the time series length and embedding dimension were optimized respectively by particle swarm optimization (PSO) algorithm. Then the fault features of rolling bearing were extracted by parameter optimized MPE and the standard clustering centers is obtained with GG clustering. Finally, the samples are clustered with the Euclid nearness degree to obtain recognition rate. The validity of the parameter optimization is proved by calculating the partition coefficient and average fuzzy entropy. Compared with unoptimized MPE, the propose method has a higher fault recognition rate.

## 1. Introduction

As the core component of rotating machinery, the state of rolling bearing directly affects the use of the equipment [1]. Vibration signals collected by the sensor are often contaminated by noise and thus unusable for direct machine faults diagnosis [2]. How to identify the state of rolling bearing quickly and effectively has become a focus of current research. Fault feature extraction and pattern recognition are key links in the fault diagnosis of rolling bearing [3,4]. At present, for the non-stationary complex signal, the feature extraction method mainly applies traditional time-frequency analysis [5] and filtering. Its statistical characteristics in time and frequency domain change with time, such as root mean square (RMS) [6], kurtosis [7], and shape factor [8]; however, these indicators will change whether the fault location occurs in the bearing outer ring, bearing inner ring or rolling element when a bearing fails. Relying solely on these eigenvalues cannot effectively distinguish and identify the fault location. Fast Fourier transform (FFT) [9], Wavelet transform [10], and ensemble empirical mode decomposition (EEMD) [11] are commonly used to signal denoising in feature extraction of fault diagnosis. Fault types are determined by comparing current fault features with standard or existing fault features [12,13]. However, due to the factors such as friction, vibration, and load in the process of mechanical operation, the vibration signal of mechanical system often shows nonlinear behavior. Using the method of time-frequency analysis to decompose the signal into stable signal inevitably has some limitations and difficulties [14].

The method of nonlinear analysis can directly extract the fault information hidden in the vibration signal of mechanical system without decomposing the original signal [15]. For the nonlinear complex signal of equipment fault, its complexity is different in different states. The complexity analysis methods commonly used in fault diagnosis are approximate entropy [16], fuzzy entropy [17], sample entropy [18], and permutation entropy [19]. However, the above methods are based on the single scale analysis of time series. Multi scale analysis [20,21] has been applied in the field of mechanical fault diagnosis because it can reflect complex features and obtain more feature information of signals. The Approximate Entropy algorithm is used to detect cracks in a rotating shaft in the reference [22]. Zheng [23] applied the concept of multi-scale fuzzy entropy (MFE) to the fault diagnosis of bearing and achieved good results. However, the calculation amount of MFE is large, and the selection of the number of characteristic parameters lacks a certain standard. The multi-scale sample (MSE) analysis was applied to the turbulence data and successfully captured two important features of the turbulent soap films [24]. However, MSE method is slow in calculation of long data, poor in real-time performance, and is greatly affected by abrupt signal. Aziz et al. [25] put forward the concept of multi-scale permutation entropy (MPE), which is used to measure the complexity and randomness of time series at different scales, to enhance the robustness. It has advantages including simple calculation, strong anti-noise ability, short time required to obtain stable system eigenvalues, and suitable for online monitoring [14]. In references [26,27,28,29,30], multi-scale permutation entropy (MPE) is applied to fault diagnosis of rolling bearing respectively. However, the above research did not study the parameters of the MPE, Table A1 of Appendix A compares the differences between their research contents and that of this paper. Because the result of multi-scale permutation entropy value is affected by its own parameters, if the parameters are set unreasonably, the best processing effect will not be achieved. Reference [31] proposed a method to determine the permutation entropy parameters based on reconstructing the optimal phase space of time series, studied the methods to determine the embedding dimension and delay time, but ignored the length of time series. In reference [14], the length of time series was determined by observing the permutation entropy of Gaussian white noise with different lengths. Although the method can achieve a certain processing effect, the number of given data length values is fixed, and it is difficult to accurately reflect the characteristic information of original signal with Gaussian white noise. Through analysis, the setting delay time t of MPE has little influence but the embedding dimension m and the length *L* of time series have a great influence on the final processing result. If the parameter setting is unreasonable, the best processing effect will not be achieved, therefore, the parameter influence analysis and optimization of the MPE are studied in this paper.

The pattern recognition can make substantive discrimination to fault types [32]. The selection of recognizer can be based on classification [33] idea or clustering [34] idea. The typical representative of classification idea is SVM [35]. However, the calculation process of SVM needs the participation of relevant existing empirical parameters, mainly including kernel parameters and penalty factors. The selection of these two parameters which play an important role in SVM is usually based on the user’s existing experience, which greatly reduces the universality of SVM model. Cluster analysis is also one of the important methods of pattern recognition. This kind of algorithm does not need difficult parameter selection process and is widely used. Fuzzy C-means (FCM) [36], Gustafson-Kessel (GK) [37], and Gath-Geva (GG) [38] are commonly used. Generally, The FCM is only applicable to the data sets with spherical distribution. GK algorithm introduces adaptive distance norm and covariance matrix, which can reflect the dispersion degree of data along any direction or subspace, but does not change the clustering state of clustering algorithm which is similar to sphere [39,40,41,42]. GG clustering algorithm is the improved result of FCM and GK clustering algorithm [38], because it measures the distance between samples by introducing fuzzy maximum likelihood estimation method and can reflect the data of different shapes and directions [43,44], which has stronger adaptability.

Based on the above reasons, this paper proposes a method which combines parameter optimized MPE and GG clustering algorithm to extract fault features and recognition pattern of rolling bearing. The effectiveness of the proposed method is verified by several rolling bearing fault experiments.

## 2. The Proposed Method

### 2.1. MPE Theory

The MPE is to calculate the permutation entropy of time series at different scales, that is to consider the characteristics of time series at multi scales. The calculation steps are as follows. For the time series $X=[x_{i},i=1,2,\cdots N]$, the coarse-grained time series $y_{j}{}^{\left(s\right)}$ are obtained by coarse-grained processing [25],

(1) $$y_{j}^{\left(s\right)}=\frac{1}{s}\sum_{i=(j-1)s+1}^{js}x_{i},\;(j=1,2,\cdots ,[N/s])$$

where *s* is the scale factor of *X* and *N* is the length of *X*.

The phase space of each coarse-grained sequence is reconstructed, the *l*_th_ reconstruction component is

(2) $$Y_{l}{}^{\left(s\right)}=\{y_{l}^{\left(s\right)},y_{l+t}^{\left(s\right)},\cdots y_{l+(m-1)t}^{\left(s\right)}\},\;(l=1,2,\cdots ,N-(m-1)t)$$

where *m* is the embedding dimension and *t* is the delay time.

By arranging the elements of each reconstruction component in ascending order, a group of corresponding symbol sequence symbol(α) can be obtained

(3) $$symbol(\alpha )=(j_{1},j_{2},\cdots j_{m}),(\alpha =1,2,\cdots \gamma ;\gamma \leq m!)$$

Calculating the PE of each coarse-grained sequence at different scales, we get the MPE of time series *X*

(4) $$H_{D}=-\sum_{\alpha =1}^{\gamma }\rho_{\alpha }\ln\rho_{\alpha }$$

where $\rho_{\alpha }$ is the probability of each symbol(α).

### 2.2. Parameter Selection for MPE

In order to analyze the general trend of a group of data, the first step is to find the mean value. However, the mean value alone cannot fully represent the overall situation of a group of data, so the skewness of the data can be obtained [45]. The smaller the absolute value of skewness is, the more reliable the value is.

The MPE value of *X* ($X=[x_{i},i=1,2,\cdots ,N]$) from all scales constitutes the sequence $H_{D}(X)$

(5) $$H_{D}(X)=\{H_{D}(1),H_{D}(2),\cdots ,H_{D}(s)\}$$

The skewness of $H_{D}(X)$ is *skew*

(6) $$skew=E{\left[H_{D}(X)-H_{D}^{ave}(X)\right]}^{3}/\left[H_{D}^{std}(X\right){]}^{3}$$

where $H_{D}^{ave}(X)$ and $H_{D}^{std}(X)$ are the average value and standard deviation of the $H_{D}(X)$, *E*(*) stands for expectation.

Therefore, this paper selects the square function of skewness as the objective function [42] to calculate the minimum value and optimize the maximum value of the F(X)

(7) $$F(X)=\frac{1}{skew^{2}+1}$$

### 2.3. Particle Swarm Optimization

Particle swarm optimization (PSO) [46] regards the individuals in the population as particles without mass and volume in the multi-dimensional search space. Each particle has its own position and velocity, in the solution space, the fitness evaluation function is used to continuously aggregate to its personal best historical position *p_best_* and the group best historical position *g_best_* in the whole field to realize the evolution of candidate solutions. 

The special memory function of PSO makes it possible to dynamically track the current search situation and adjust its search strategy. The evolution process of particle swarm optimization is as follows

(8) $$\left\{ \begin{array}{l} ve_{i}^{\sigma +1}=w\cdot ve_{i}^{\sigma }+c_{1}r_{1}(p_{i}^{\sigma }-po_{i}^{\sigma })+c_{2}r_{2}(g_{i}^{\sigma }-po_{i}^{\sigma })\\ po_{i}^{\sigma +1}=po_{i}^{\sigma }+ve_{i}^{\sigma +1} \end{array}\right.$$

where σ is an evolutionary algebra, $ve_{i}{}^{\sigma }$ is the flight velocity of particle *i*, $po_{i}{}^{\sigma }$ is the position vector of particle *i*, $p_{i}^{\sigma }$ is the best position experienced by particle *i* and $g_{i}^{\sigma }$ is the best position of the whole particle swarm to experience in the solution space. $r_{1}$ and $r_{2}$ are random numbers between [0, 1], $c_{1}$ and $c_{2}$ are learning factors, *w* is the inertia weight factor. While $po_{i}$ and $ve_{i}$ meet the following condition,

(9) $$\left\{ \begin{array}{l} po_{i}\in [po_{\min},po_{\max}]\\ ve_{i}\in [ve_{\min},ve_{\max}]\\ ve_{\max}=\delta po_{\max} \end{array}\right.$$

where δ is the proportional coefficient between the maximum velocity $ve_{\max}$ and the maximum search space $po_{\max}$.

When the position or velocity of a certain dimensional variable exceeds the boundary range, the boundary absorption strategy is adopted, that is, the particle falls on the boundary of the search space in the next iteration.

**Figure 1:**
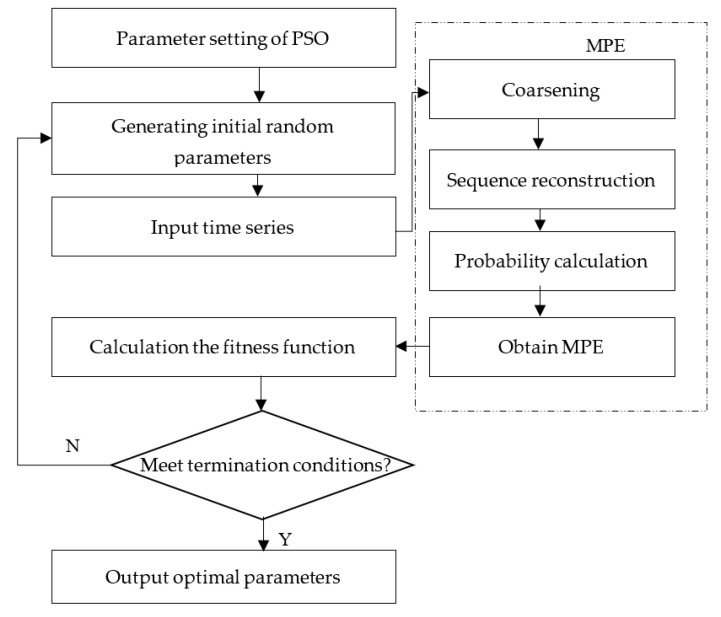
Parameter optimization procedure of MPE based on PSO.

The parameters of PSO algorithm in this paper are set as follows: population size *group* = 20, maximum iterations *T*_max_ = 10, acceleration constant $c_{1,2}$ = 1.5, and inertia weight *w* = 0.5. The process of MPE parameter optimization using PSO is shown in Figure 1.

### 2.4. GG Algorithm

The specific algorithm given in the reference [47] is as follows.

(1) Suppose a sample set $\Omega =(\psi_{1},\psi_{2},\cdots \psi_{k},\cdots \psi_{n})$ has a z(2≤z≤n) common class, $\psi_{k}=[\psi_{k1},\psi_{k2},\cdots ,\psi_{kd}]$ representing *d* features of the $k_{\mathrm{th}}(1\leq k\leq n)$ sample.

(2) Initialize membership matrix $U={\left[u_{ik}\right]}_{z\times n}$, $u_{ik}$ is objective function, which indicates the degree of the *k*_th_ sample belonging to the *i*_th_ (1≤i≤z) category. $V=[v_{1},v_{2},\cdots ,v_{z}]$ is cluster center vector, *z* is the number of clusters. 

(3) Update cluster center $v_{i}$

(10) $$v_{i}^{\lambda }=\frac{\sum_{k=1}^{n}{\left(u_{{}_{ik}}^{\lambda -1}\right)}^{\beta }\psi_{k}}{\sum_{k=1}^{n}{\left(u_{{}_{ik}}^{\lambda -1}\right)}^{\beta }}$$

where λ is the iterations, β is fuzzy exponent and generally taken as 2.

(4) Calculate the distance measure $DM_{ik}$

(11) $$DM_{ik}=\frac{{\left(\det(A_{i})\right)}^{1/2}}{q_{i}}\exp(\frac{1}{2}{\left(\psi_{k}-v_{i}^{\lambda }\right)}^{T}A_{i}^{-1}(\psi_{k}-v_{i}^{\lambda }))$$

(12) $$q_{i}=\frac{1}{n}\sum_{k=1}^{n}u_{ik}$$

where $DM_{ik}$ is the maximum likelihood estimation distance and $A_{i}$ is the covariance matrix of the *i*_th_ cluster center, $q_{i}$ is the prior probability of the *i*_th_ cluster being selected.

(5) Update membership matrix *U*

(13) $$u_{{}_{ik}}^{\lambda }=\frac{\sum_{k=1}^{n}u_{{}_{ik}}^{\lambda -1}}{\sum_{j=1}^{z}{\left(DM_{ik}(\psi_{k},v_{i})/DM_{jk}(\psi_{k},v_{j})\right)}^{\frac{2}{\beta -1}}}$$

where if the condition $\left\|U^{\lambda }-U^{\lambda -1}\right\|<\varepsilon$ (ε is the termination tolerance) is satisfied, the operation will be terminated, otherwise, λ=λ+1, until the condition is satisfied.

### 2.5. Evaluation Index of Clustering Effect

The clustering effect of GG fuzzy clustering can be made quantitative assessment with partition coefficient (*PAC*) [48] and partition entropy (*PAE*), which are as follows

(14) $$PAC=\frac{1}{n}\sum_{i=1}^{z}\sum_{k=1}^{n}u_{{}_{ik}}^{2}$$

(15) $$PAE=-\sum_{i=1}^{z}\frac{\zeta_{i}}{n}(\sum_{\tau =1}^{\ell }\frac{\zeta_{i\tau }}{\zeta_{i}}\log_{2}\frac{\zeta_{i\tau }}{\zeta_{i}})$$

where $\zeta_{i}$, $\zeta_{i\tau }$ are the number of all members in cluster *i* and the number of members belonging to class τ, respectively. ℓ is the number of category from cluster *i*.

Suppose the sample set $\Omega =(\psi_{1},\psi_{2},\cdots \psi_{k},\cdots \psi_{n})$ is composed of the sample set called θ and the set φ, *n* is the number of samples in Ω, Euclidean closeness [49] is used to fault recognition in this paper, then the Euclid closeness between θ and φ is

(16) $$Euclid(\theta ,\varphi )=1-\frac{1}{\sqrt{n}}\sqrt{\sum_{k=1}^{n}{\left[\theta (\psi_{k})-\varphi (\psi_{k})\right]}^{2}}$$

where $\theta (\psi_{k})$ and $\varphi (\psi_{k})$ are membership functions of θ and φ, respectively.

### 2.6. The Process of Bearing Fault Pattern Recognition

**Figure 2:**
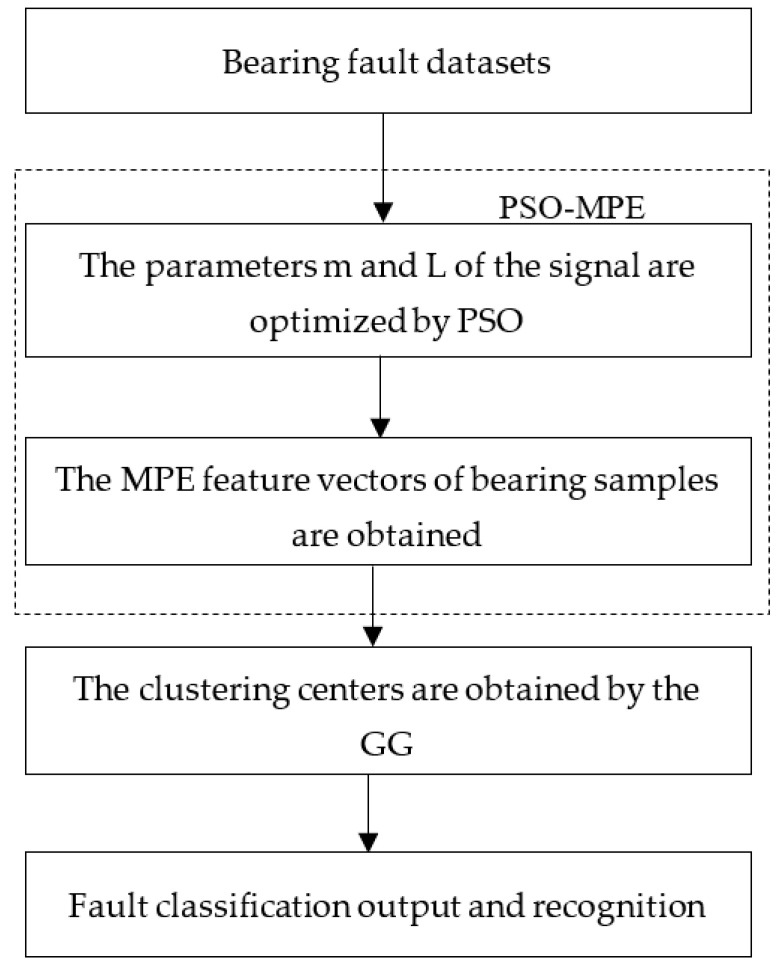
Implementation flow chart of proposed method.

The framework of the proposed method is shown in Figure 2. The general implementation procedure is summarized as follows

(1) Carry out the experiment and collect the vibration experiment data.
(2) For the signal, the initial parameters of MPE are optimized by PSO algorithm. The optimal parameters (*m*, *L*) of MPE is determined.
(3) The optimized parameters are reset to MPE, the entropy of signal is calculated by PSO-MPE and the eigenvalue matrix is established.
(4) Input eigenvalue matrix into GG clustering classifier to realize clustering.
(5) The Euclidean distance between the samples to be identified and the clustering center is calculated to realize the classification and recognition.

## 3. Parameter Influence Analysis of MPE

**Figure 3:**
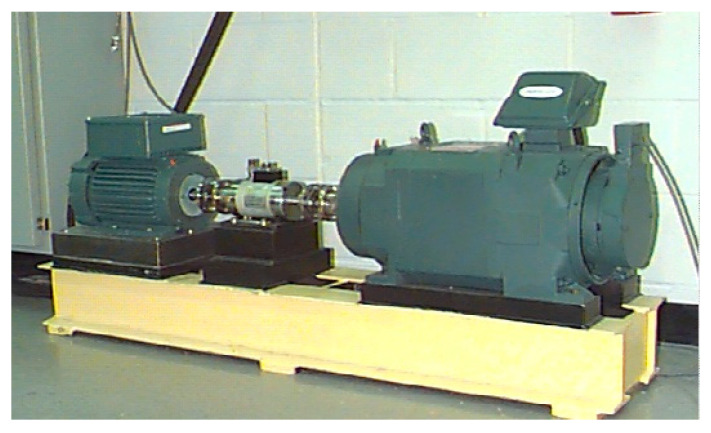
The test stand of CWRU.

In order to study the influence of different parameters on MPE, the experimental data of rolling bearing in Case Western Reserve University [50] is used for analysis. The test stand is shown in Figure 3, which is composed of a 2 hp motor (left), a torque transducer/encoder (center), a dynamometer (right), and control electronics (not shown). The test bearings support the motor shaft. Single point faults were introduced to the test bearings using electro-discharge machine. Vibration data was collected using accelerometers, which were attached to the housing with magnetic bases. The rolling bearing near the drive end is tested in the experiment. Its type is 6205-2 RSJEMSKF.

**Figure 4:**
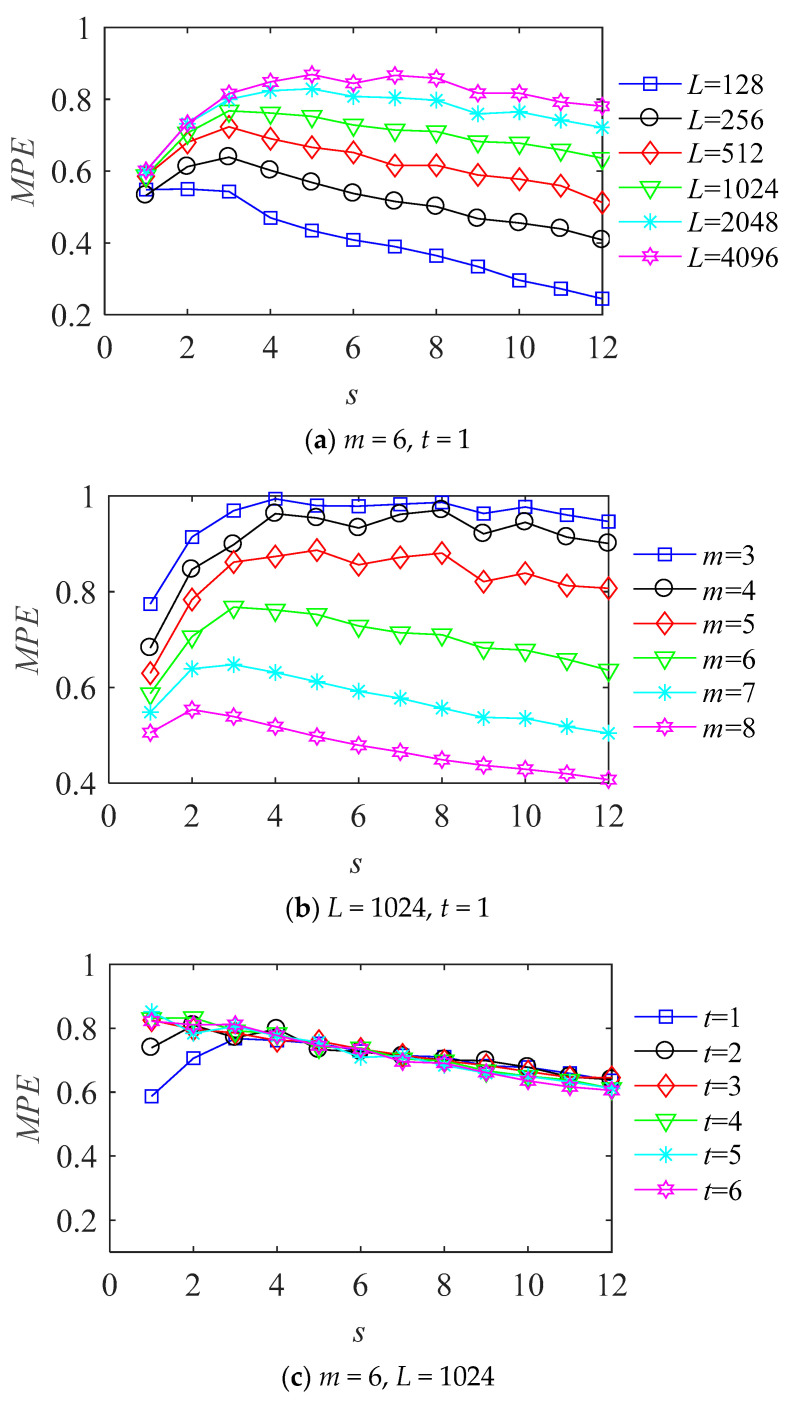
The multi-scale permutation entropy of different parameters.

Taking the normal vibration signal of the drive end bearing as an example when the motor speed is 1797 r/min, the sampling frequency is 12 kHz. The values of data length *L* are 128–4096, respectively. The values of embedding dimension *m* are 3–8, delay time *t* is 1–6 and scale factor *s* is set from 1 to 12. Figure 4 shows the amplitude variation of MPE from samples in each state under different lengths, different embedding dimensions, and different delay time.

It can be seen from Figure 4 that for the normal vibration signal of the bearing, when *m* = 6, *t* = 1, the value of *L* changes from small to large, the entropy increases obviously. So different *L* values have a greater impact on the entropy, it is necessary to select the appropriate value of *L*. Fixed *L* = 1024, *t* = 1, *m* values from small to large change, with the increase of *m*, the entropy decreases obviously, different m value has a different entropy, so it is necessary to select the appropriate value of *m*.

Fixed *m* = 6, *L* = 1024, as can be seen from Figure 4c, with the increase of delay time *t*, the entropy value does not increase or decrease obviously at different scales, which indicates that it has little effect on the entropy value, so the fixed value of *t* is 1 in this paper. When *m* value is too small, the ability of the algorithm to detect signal mutation is low, but the larger *m* value is, the larger the amount of calculation is, and the longer the running time of the algorithm is. In summary, selecting the appropriate data length *L* and embedding dimension *m* is necessary.

## 4. Experimental and Comparative Analysis

### 4.1. Case 1: CWRU Data Analysis

**Figure 5:**
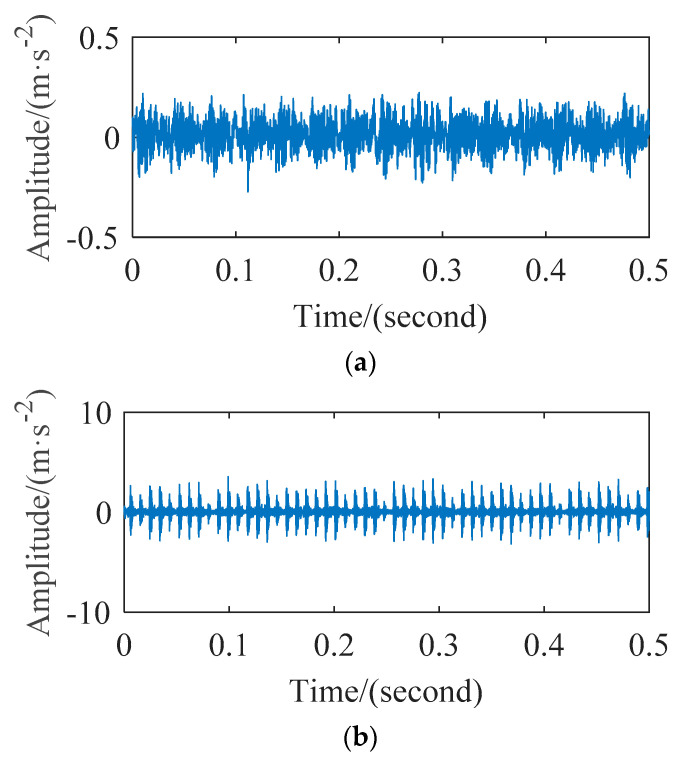

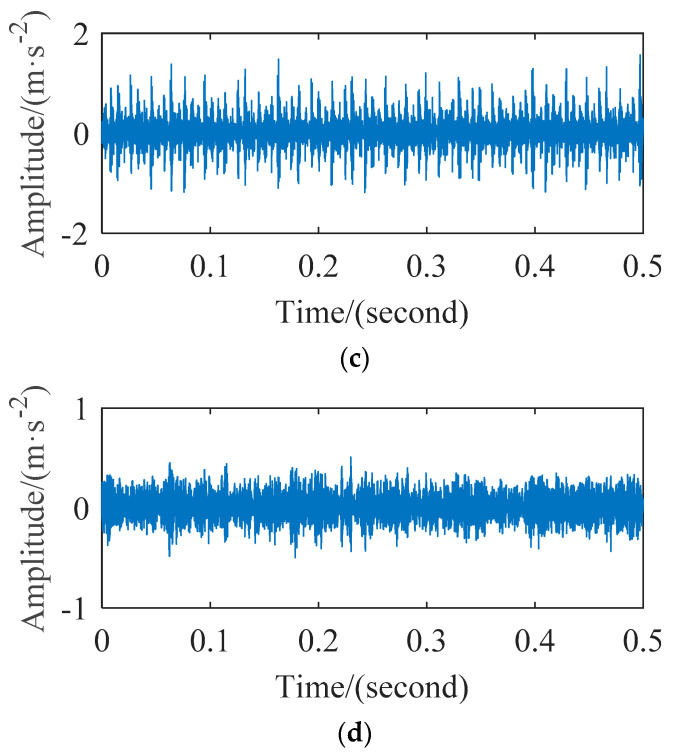
The waveforms of the vibration signal: (**a**) normal; (**b**) outer race fault; (**c**) inner race fault; (**d**) ball fault.

When the motor speed is 1797 r/min, four types of vibration signals are analyzed, including normal (NR) bearings, outer ring fault (ORF) bearings, inner ring fault (IRF) bearings and ball fault (BF) bearings. Figure 5 shows a part of time waveform of the vibration signal collected by sensors in four states, the horizontal axis is the time, the vertical axis is the acceleration amplitude of the vibration signals, their units are second and $m\cdot s^{-2}$ respectively. Intercept each state signals from the original signal according to different lengths to obtain four state samples. The number of samples is 30 for each state, a total of 120 feature vectors can be obtained, and each feature vector has 12 dimensions.

**Figure 6:**
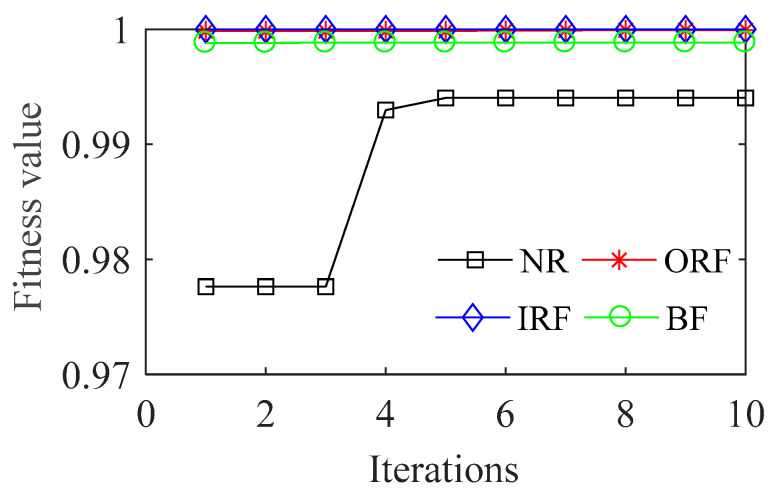
The iterative process.

Firstly, every sample is analyzed by MPE to extract the features, the effectiveness of parameter optimization of PSO algorithm is verified compared with the parameters in reference [32]. The vibration signals of four states of the bearing are analyzed, and the change of fitness value in the optimization process is shown in Figure 6.

**Figure 7:**
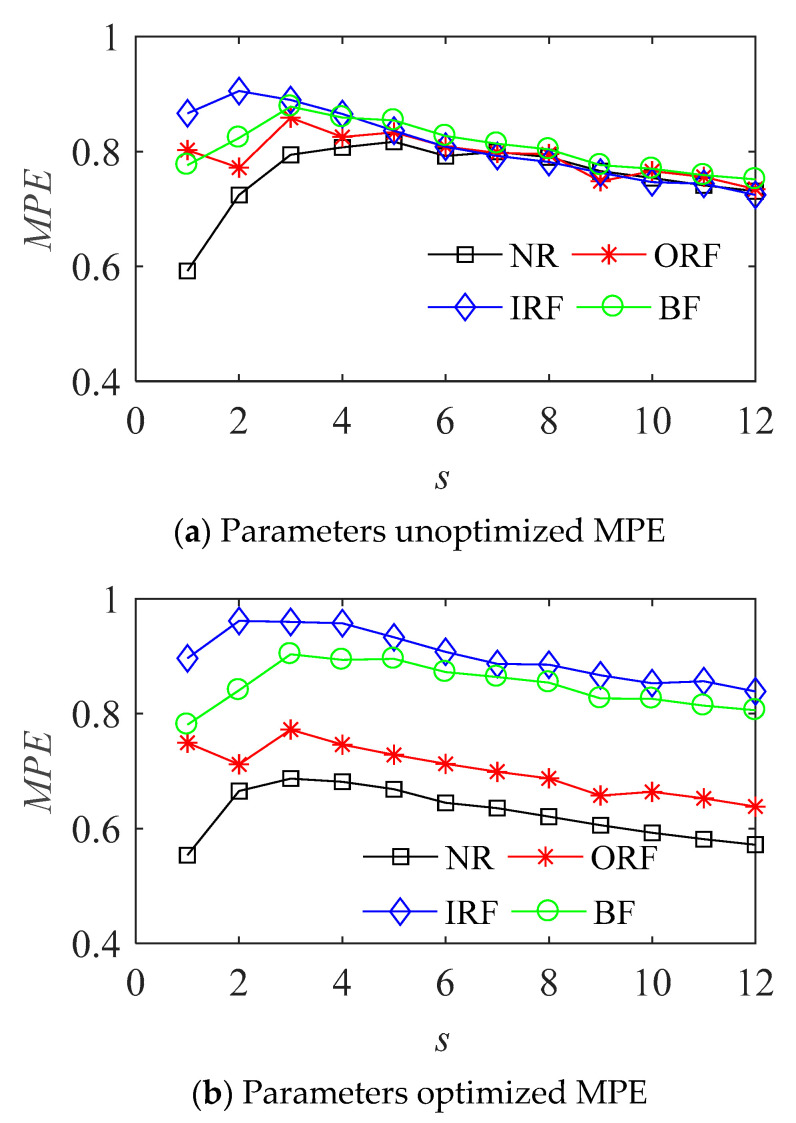
The MPE of bearing vibration signals in four states.

**Figure 8:**
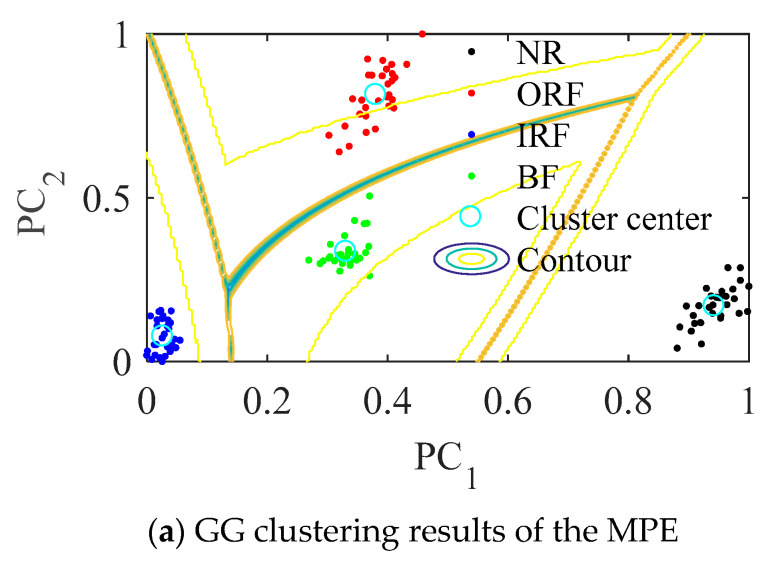

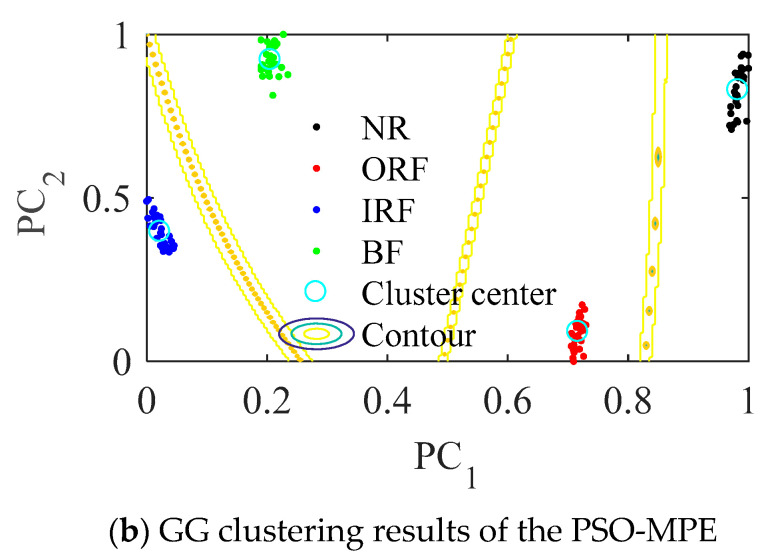
The clustering results of bearing vibration signals in four states.

The optimized parameters of MPE for various state samples are shown in Table 1. The MPE values and cluster results before and after the optimization of vibration signal in four states are shown in the Figure 7 and Figure 8 respectively.

It can be seen from Figure 7b that the MPE with optimized parameters can better distinguish the four different states of bearings, and is better than the effect of fixed parameters in Figure 7a. The parameter optimized MPE can distinguish the four states of the bearing more obviously, it can be used as the feature vector to further classify and identify the bearing fault modes.

In Figure 8, ${\mathrm{PC}}_{1}$ and ${\mathrm{PC}}_{2}$ are two vectors in two-dimensional space after data visualization, they have the same meaning in the following similar figures. As can be seen from Figure 8b, the samples are distributed around four clustering centers according to fault types after processed by the proposed method, the distance between different classes becomes larger and the distance within classes becomes smaller compared with Figure 8a.

In order to further illustrate the effectiveness of this research method, the *PAC*, *PAE,* and fault recognition rate are used to evaluate quantitatively. Corresponding to Figure 8, the performance comparison of the two recognition methods is shown in Table 2. It can be seen that

(1) The closer the *PAC* is to 0, the better the clustering effect. Although the *PAC* value of MPE and PSO-MPE are all 1, the *PAE* decreases gradually. The closer the *PAE* is to 0, the better the clustering effect.
(2) The fault recognition rate of PSO-MPE with GG clustering reaches 100%, which is consistent with its clustering performance.
(3) It can be seen that the PSO-MPE method proposed by the author can effectively extract the fault feature information of rolling bearing and accurately identify different fault types of rolling bearings.

### 4.2. Case 2: A Freight Locomotive Wheelset Bearing Signal

**Figure 9:**
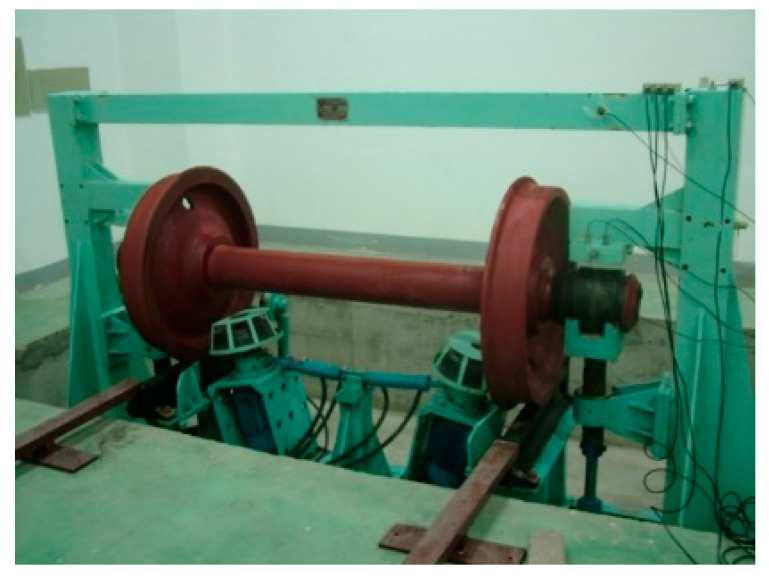
Experimental setup.

**Figure 10:**
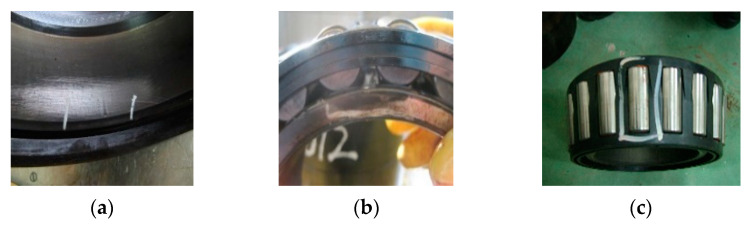
The fault locations: (**a**) The slight dent fault on the outer raceway; (**b**) a fatigue spall fault on the inner raceway; (**c**) the scratch fault on the rolling ball.

To further demonstrate the performance of the proposed method, a fault experiment is carried out in this section. The experimental setup and the tested wheelset bearing are shown in Figure 9. RD2 wheel set and 197,726 double row tapered roller bearing are installed on the test bench. The fault bearings are shown in Figure 10. The wheelset bearing defections are natural damages generated during the operation of the railway freight vehicles, which are located in the outer raceway, inner raceway and ball, respectively. The experimental device includes three DASP data processing software of CA-YD-188 piezoelectric accelerometer, signal amplifier and INV36DF signal acquisition instrument. The sensors are installed on the test bench in turn, and the position is shown in Figure 9. The sampling frequency is 25.6 kHz. 

**Figure 11:**
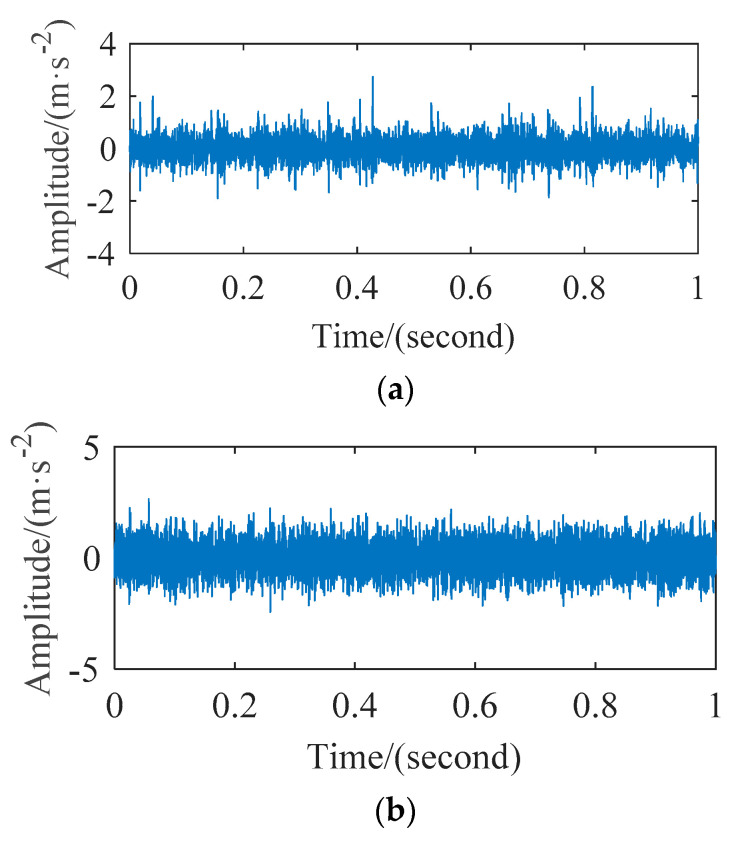
The time-domain waveforms: (**a**) Inner raceway fault; (**b**) ball fault.

In order to observe the time domain characteristics and save the paper space, Figure 11 shows the time domain waveform of the bearing inner ring and rolling ball. We can see the noise component of the collected signal from this experiment in the Figure 11 is more than bearing experiment of CWRU in the Figure 5, which increases difficulty of the method verification.

**Figure 12:**
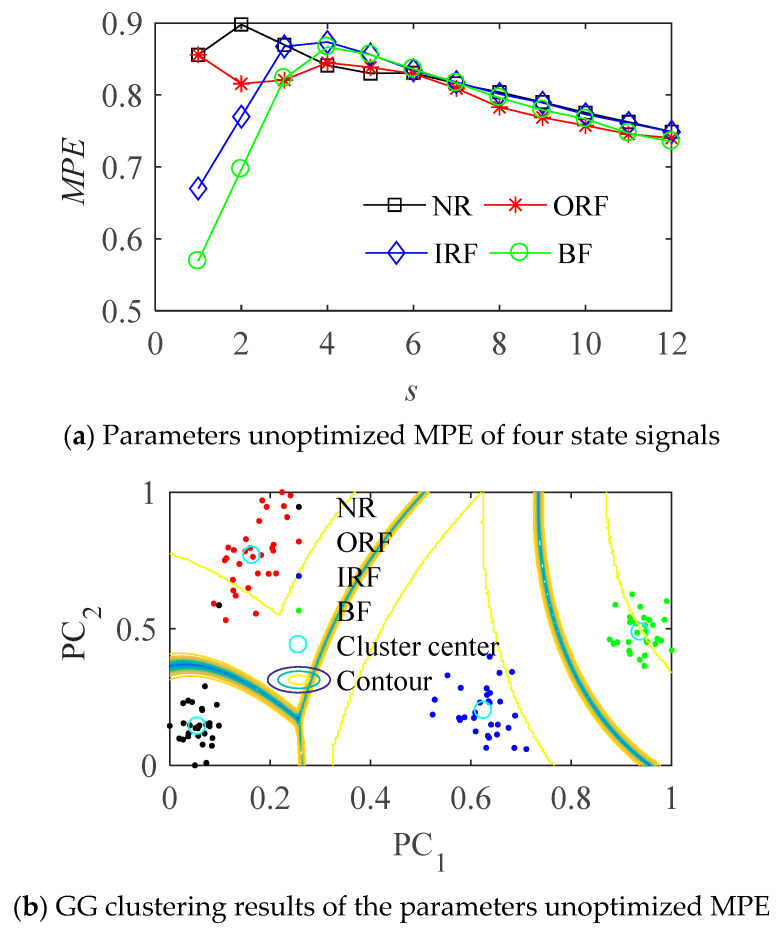
The results with MPE.

There are 30 group samples collected in each state. It can be seen from Figure 12 that without optimizing the parameters of the MPE, the entropy values of the four states of rolling bearing are intertwined, they are not effectively distinguished, which cannot effectively distinguish the four states, it is not suitable to use them as the quantitative features of rolling bearing fault.

GG with parameters unoptimized MPE is directly used for the signal. As shown in Figure 12a, the entropy value of the four states is not effectively distinguished. The sample distance of the same class is too large, and the distance between different classes is small in Figure 12b. Although we can see about four gathering teams, the distinction between NR and ORF is not obvious, some NR samples are wrongly classified into ORF, when the signal contains more noise components, it is easy to misjudge.

**Figure 13:**
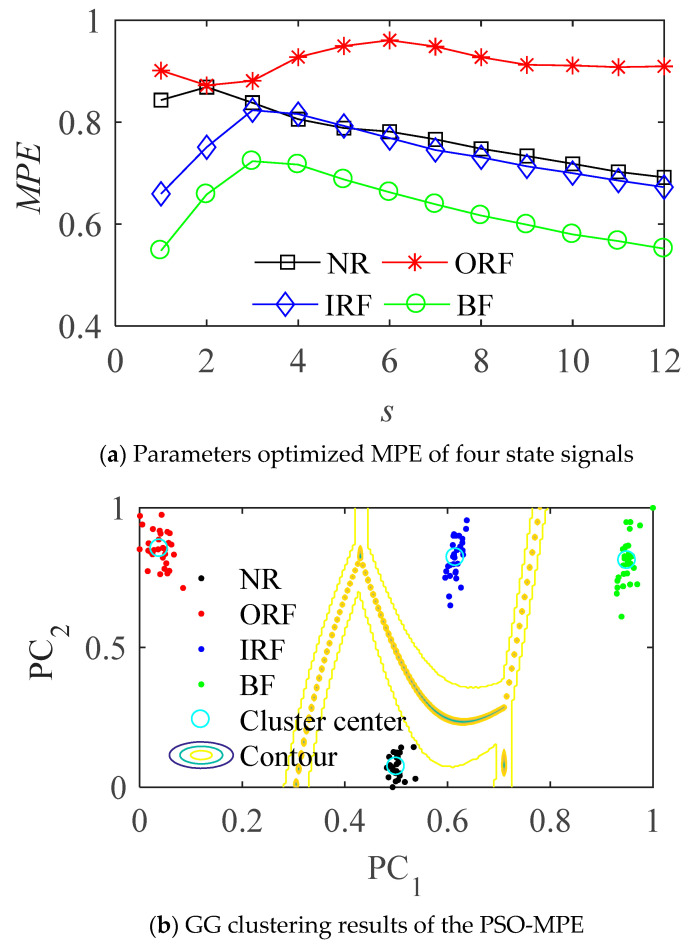
The results with PSO-MPE.

The Table 3 are the parameters of MPE in various states obtained by PSO. Figure 13a shows the PSO-MPE of four state signals, it can be seen that distance between the entropy curves of different operation states is significantly increased and entropy curves of different operation states are obviously separated completely. This is because when the rolling bearing has faults, the randomness of vibration signal changes, which changes the entropy values in different scales. In the same state, with the increase of scale, the randomness and complexity of coarse-grained sequence decrease, and the change range of entropy decreases.

As can be seen from Figure 13b, after samples are processed by PSO-MPE and GG clustering algorithm, they are distributed around four clustering centers according to fault types, the distance between different classes becomes larger and the distance within class becomes smaller than Figure 13a.

According to Table 4, The fault recognition rate of rolling bearing based on PSO-MPE and GG clustering is 99.17%, which is higher than the recognition rate of MPE. Moreover, the *PAC* and *PAE* are better than those of parameters unoptimized MPE. It shows that the proposed method is still effective under relatively difficult experimental conditions.

**Figure 14:**
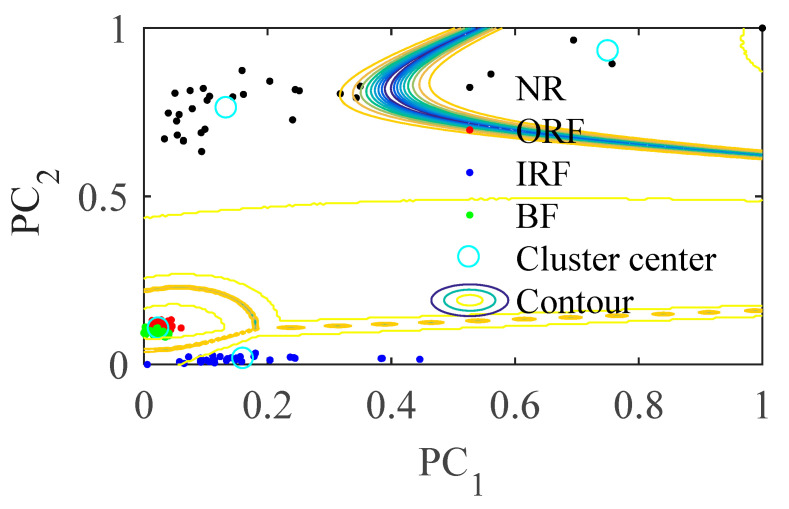
The GG clustering results with Kurtosis and RMS.

In order to prove the superiority of parameter optimized MPE as signal feature extraction index, compare it with the feature vector composed of kurtosis and root mean square. Figure 14 shows the effect of clustering with kurtosis and root mean square (RMS) as feature vector. Compared with the Figure 13b, it is obvious that the four types of samples are not effectively distinguished, because these indexes will change no matter which part of the bearing fails, it cannot effectively distinguish the fault location only through kurtosis or root mean square. While the research method in this paper can effectively distinguish different types of fault samples.

### 4.3. Case 3: A High-Speed Locomotive Wheelset Bearing Fault Signal

**Figure 15:**
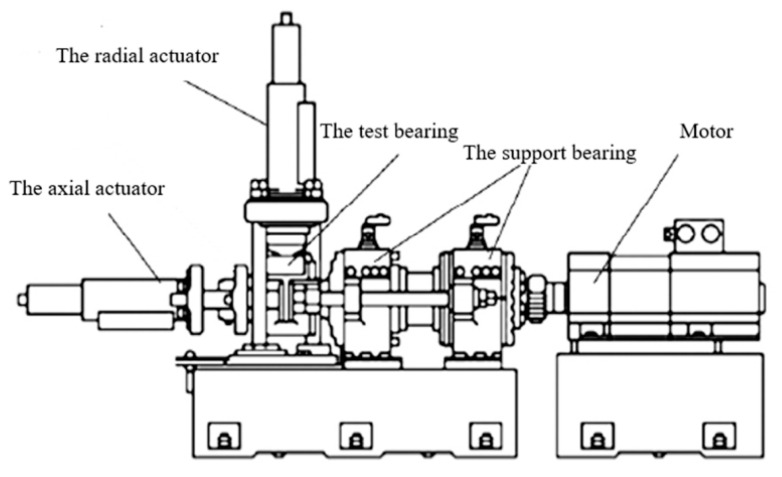
The structure of the test rig.

In order to verify whether the method is still effective in more complex working conditions with more noise components, the practical test data from the self-made experiment platform is selected for subsequent analysis. In this case, the vibration signal has been collected from a high-speed locomotive wheelset bearing. The test rig structure [51] is depicted in Figure 15. In order to simulate the load change of wheel set bearing during operation, apply a random force with a frequency of 0.2~20 Hz and an average value of about 10 kN in the radial direction, a simple harmonic force with a frequency of 1 Hz and a maximum value of 10 kN is applied axially on the test rig.

**Figure 16:**
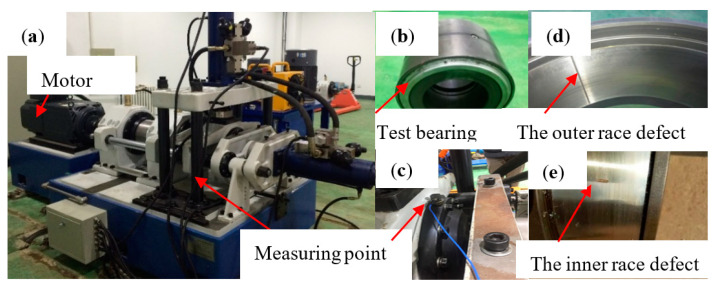
Railway bearing test bench and bearings: (**a**) Railway bearing test bench overview; (**b**) test bearing; (**c**) local enlarged test point; (**d**) partial enlarged view of outer race fault; (**e**) partial enlarged view of inner race fault.

The field diagram of the test rig and the test bearings are depicted in Figure 16. The sensor is located at the top of the end-shield of the test bearing in Figure 16c and the vibration signal is collected by a PCB356A25 accelerometer. The dynamic loads can be obtained by the radical and axial actuators. There is an artificial local defect in the outer race of test bearing as plotted in Figure 16d, of which the width is 1 mm and length is 5 mm. It can be noted that the artificial defect is relatively slight in comparison with its geometries. The sampling frequency is set as 51.2 kHz and the set speed is 2000 r/min. 

**Figure 17:**
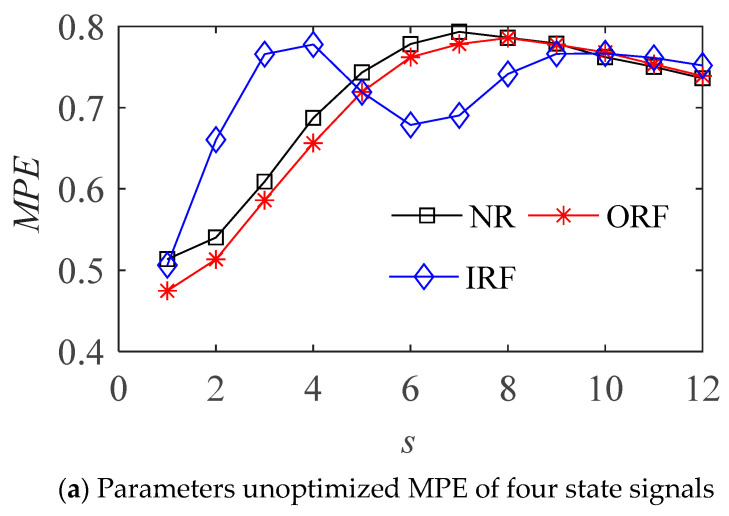

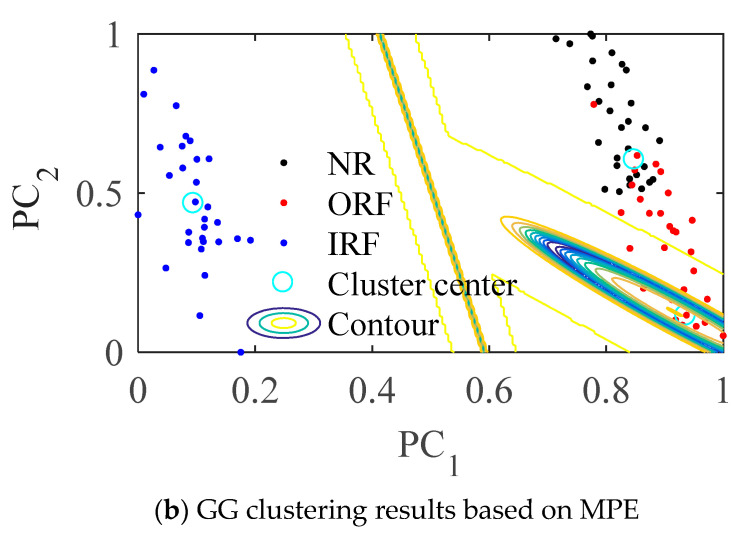
The results with MPE.

Each state collected 30 samples. There were total three kinds of normal signals and vibration signals at different fault positions. The results solved by the MPE are shown in the Figure 17 when the *L* = 2048, *m* = 6.

As can be seen from Figure 17, GG clustering cannot effectively cluster the fault feature samples constructed by MPE. It is difficult for the entropy to represent the different running states of bearings so further treatment is necessary. The steps are the same as last section, it will not be repeated here.

**Figure 18:**
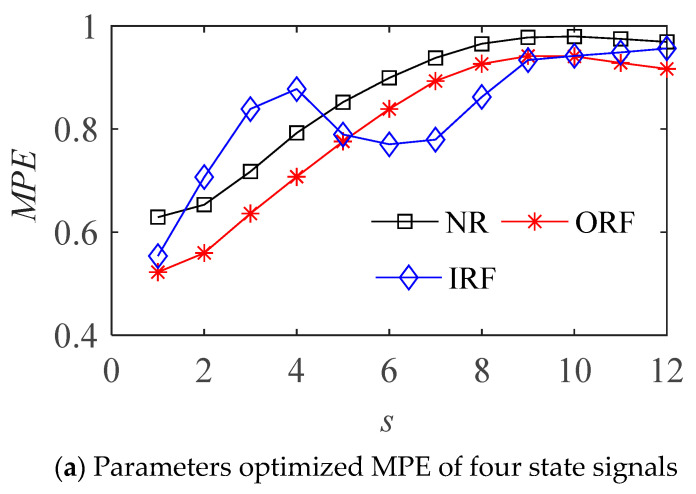

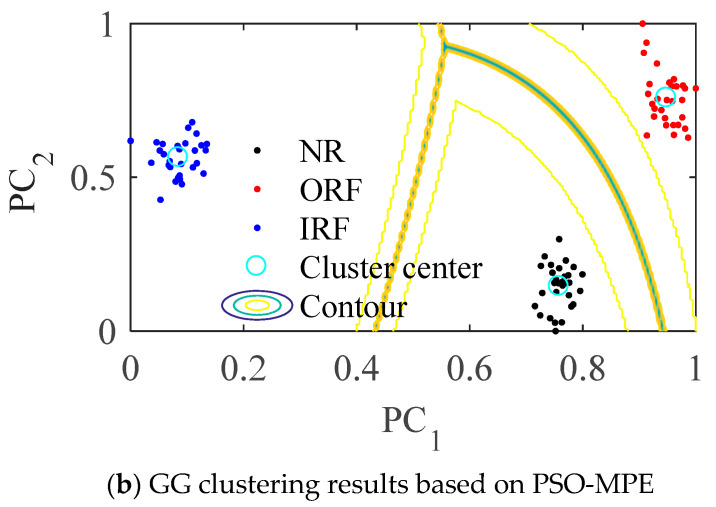
The results with PSO-MPE.

The Table 5 are the parameters of MPE in various states, which are obtained by PSO algorithm. Then GG is used to cluster the samples. The results with PSO-MPE are show in Figure 18.

It can be seen from Figure 18a, the proposed method can effectively distinguish the three states. The values of NR and ORF is obviously separated while they are not in Figure 17a. Compared with Figure 17b, the samples of each state in Figure 18b are obviously separated, classified with its own cluster centers and the distance between different classes becomes larger and the distance within classes becomes smaller, respectively.

According to Table 6, The fault recognition rate of rolling bearing based on the proposed method is 100%, which improves a lot than the 78.89% of the MPE. Moreover, the *PAC* and *PAE* of PSO-MPE are better than those of MPE, which prove the necessity and advantage of combination PSO-MPE and GG method, it has better clustering effect and recognition effect.

**Figure 19:**
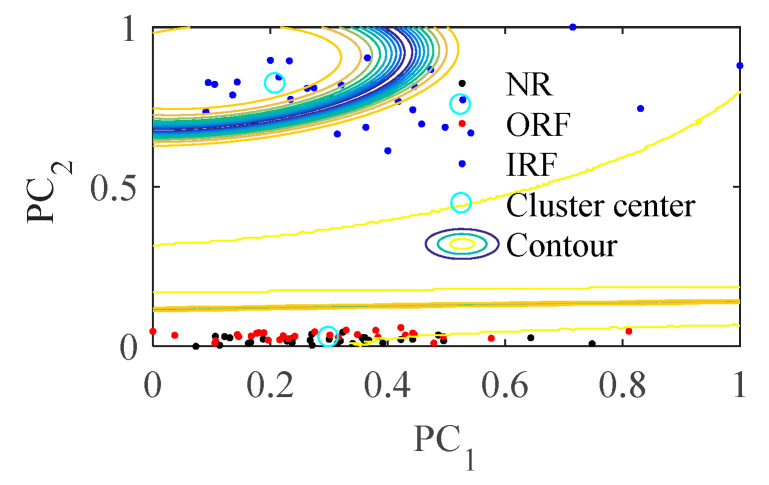
The GG clustering results with Kurtosis and RMS.

In order to prove the robustness advantage of the proposed method, compare it with the feature vector composed of kurtosis and root mean square (RMS). Figure 19 shows the clustering effect of kurtosis and root mean square as feature vector. Compared with the Figure 18b, it is obvious that the three types of samples are not effectively distinguished, because the experimental environment simulates the working condition of high-speed train operation, the collected signal is close to the vibration signal of the train running on the actual line and is seriously disturbed by environmental noise. This index is almost invalid in this case.

## 5. Conclusions

In this paper, a rolling bearing fault detection method based on the PSO-MPE and GG is proposed. The method can select the important parameters of MPE method adaptively, overcome the disadvantages of fixed MPE parameters and greatly improve the accuracy of fault identification. The method is verified by several experiments. Some conclusions are obtained as follows:

(1) To solve the problem of parameter determination of MPE, fitness function is constructed by skewness of multi-scale permutation entropy, the time series length *L* and embedding dimension *m* are optimized, the effectiveness of the optimization method is verified by experiments.

(2) Compared with the MPE of fixed parameters, it is proved that parameter optimized MPE can extract fault features accurately and has better classification and recognition rate about the rolling bearing typical faults.

(3) The effectiveness and robustness of the proposed method is verified by several rolling bearing experiments, of which the signals are simple to complex. Meanwhile, compared with the feature vector composed of root mean square and kurtosis, the proposed method shows advantages when the vibration signal contains more noise components and serious environmental interference, the proposed method has more accurate and stable performance in fault diagnosis.

## Figures and Tables

**Table 1:** The parameters of MPE.

Fault Types	Parameters of MPE from Reference [32]		Parameters of MPE with PSO	
	*m*	*L*	*m*	*L*
NR	6	2048	7	1732
ORF	6	2048	7	3152
IRF	6	2048	5	1559
BF	6	2048	6	3251

**Table 3:** The PSO-MPE parameters.

Fault Types	*m*	*L*
NR	6	1339
ORF	5	3180
IRF	6	1168
BF	6	541

**Table 4:** Performance comparison of two recognition methods.

Classifier	Evaluating Indicators	MPE	PSO-MPE
GG	*PAC*	0.9999	1
	*PAE*	0.3658	0.0409
	Recognition rate	81.67%	99.17%

**Table 5:** The PSO-MPE parameters.

Fault Types	*m*	*L*
NR	4	3818
ORF	5	3745
IRF	5	3918

**Table 6:** Performance comparison of two recognition methods.

Classifier	Evaluating Indicators	MPE	PSO-MPE
GG	*PAC*	0.9841	1
	*PAE*	0.2720	0
	Recognition rate	78.89%	100%

**Table 2:** Performance comparison of two recognition methods.

Classifier	Evaluating Indicators	MPE	PSO-MPE
GG	*PAC*	1	1
	*PAE*	0.2624	0
	Recognition rate	84.17%	100%

## Data Availability

The data are not publicly available due to laboratory restrictions.

## Appendix A

**Table A1:** Similarities and Differences between This Paper and Other References.

References	Similarities	Differences
Reference [26]: LMD-MPE	The MPE of vibration signal is extracted as the feature for fault subsequent classification or clustering	The local mean decomposition (LMD) is used to denoise, then calculate MPE of the product functions, SVM is used to classify. LMD-MPE and LMD-MPE are compared to prove the advantages of MPE
Reference [27]: VMD-MPE-FCM		The variational mode decomposition (VMD) is used to denoise and obtain intrinsic mode functions (IMF), calculate MPE of these IMFs, FCM is used to cluster. The parameters influence of MPE is not considered.
Reference [28]: MPE-LS-SVM		The MPE of the bearing vibration signal in different scales is calculated, the Laplacian score (LS) is used to refine the feature vector, SVM is used to classify. The parameters of MPE is fixed.
Reference [29]: EEMD-MPE-SA-SVM		A number of intrinsic mode functions (IMFs) are obtained using ensemble empirical mode decompose (EEMD), the multi-scale IMF permutation entropy are extracted, SA-SVM is used to classify. The parameters (*m*,*t*) of MPE is fixed.
Reference [30]: CEEMD-MPE-GK		Complementary Ensemble Empirical Mode Decomposition (CEEMD) is used to denoise and obtain intrinsic mode functions (IMF), calculate MPE of the modal IMF, the GK is used to fault type recognition. The parameters influence of MPE is not considered.
This paper: PSO-MPE-GG		The embedding dimension *m*, delay time *t* and the length *L* of time series sample are considered when calculate MPE, PSO and skewness are used to determine the selection of parameters, which increases the robustness of the MPE. GG is used for cluster of fault types, which increases the accuracy.
